# Adipose Derived Stem Cells Exert Immunomodulatory Effects on Natural Killer Cells in Breast Cancer

**Published:** 2016-12-21

**Authors:** Behdokht Bahrami, Ahmad Hosseini, Abdol-Rasoul Talei, Abbas Ghaderi, Mahboobeh Razmkhah

**Affiliations:** 1 Shiraz Institute for Cancer Research, School of Medicine, Shiraz University of Medical Sciences, Shiraz, Iran; 2 Department of Immunology, School of Medicine, Shiraz University of Medical Sciences, Shiraz, Iran; 3 Breast Diseases Research Center (BDRC), Shiraz University of Medical Sciences, Shiraz, Iran

**Keywords:** Mesenchymal Stem Cells, Immunosuppression, NK Cells, Breast Cancer

## Abstract

**Objective:**

Adipose derived stem cells (ASCs), as one of the important stromal cells in the tumor microenvironment, are determined with immunomodulatory effects. The principle aim of this study was to evaluate the immunosuppressive effects of ASCs on natural killer (NK) cells.

**Materials and Methods:**

In this experimental study, we assessed the expressions of indolamine 2, 3-dioxygenase (*IDO1*), *IDO2* and human leukocyte antigen-G5 (*HLA-G5*) in ASCs isolated from breast cancer patients with different stages as well as normal individuals, using quantitative reverse transcriptase-polymerase chain reaction (qRT-PCR). Immunomodulatory effects of ASCs on the expression of CD16, CD56, CD69, NKG2D, NKp30, NKG2A and NKp44 was also assessed in peripheral blood lymphocytes (PBLs) by flow-cytometry.

**Results:**

Our result showed that *IDO1*, *IDO2* and *HLA-G5* had higher mRNA expressions in ASCs isolated from breast cancer patients than those from normal individuals (P>0.05). mRNA expression of these molecules were higher in ASCs isolated from breast cancer patients with stage III tumors than those with stage II. The indirect culture of ASCs isolated from breast cancer patients and normal individuals with activated PBLs significantly reduced NKG2D+ and CD69+ NK cells (P<0.05).

**Conclusion:**

Results of the present study suggest more evidences for the immunosuppression of ASCs on NK cells, providing conditions in favor of tumor immune evasion.

## Introduction

It has long been indicated that in spite of antitumor immune responses, malignant cells may not be completely eliminated from body. This process may be explained by the gradual changes in the nature of the anti-tumor immunity from proinflammatory to anti-inflammatory responses (1,2). The exact mechanisms underlying this process have not been clearly known; however many studies agree on the role of various immune and stromal cells such as mesenchymal stem cells (MSCs) which are present in the tumor microenvironment. MSCs may have crucial roles in this process, due to the release of a number of cytokines and regulatory molecules such as interleukin-6 (IL-6), IL-8, transforming growth factor-beta1 (TGF-β1), IDO, stromal derived facto-alpha (SDF-1α) and IL1β (3,7). Tumor cells under special circumstances, such as hypoxia, have extensive ability to motivate and trigger MSCs through secretion of IL-6 and monocyte chemoattractant protein-1 (MCP^1^) (4,8). Furthermore, MSCs can differentiate into cancer-associated fibroblasts (CAFs) that produce high levels of IL-4, IL-10, TGF-β1 and vascular endothelial growth factor (VEGF) and regulate epithelial-mesenchymal transition (EMT) mechanism, thus they support cancer cell survival and metastasis (9,10). MSCs may be considered as the main origin of myofibroblasts in tumor sites such as breast cancer microenvironment, which could likely protect tumor cells from the host immune responses, resulting in tumor growth and invasion (11). 

A plenty of studies showed the inhibitory effects of MSCs on the maturation, pro-inflammatory potential and differentiation of B cells, dendritic cells (DCs), NK cells and cytotoxic T-cells (2,12,17). MSCs can inhibit proliferation, interferongamma (IFN-γ) production and cytotoxic activity of natural-killer (NK) cells by down-regulating the expression of activating receptors such as natural cytotoxicity receptor 3 (NKp30 or CD337) and natural-killer group 2, member D (NKG2D or CD314), and through production of prostaglandin E2 (PGE2), IDO and soluble human leukocyte antigen-G5 (sHLA-G5) (18). These cells have an ability to inhibit NK cell functions by decrease in intracellular and secreted granzyme B (19). 

Adipose tissue is the most abundant stromal tissue in breast. There is little information about the role of adipose derived stem cells (ASCs), as one the mesenchymal stem cells, in the process of breast tumor growth. In addition, cross talk between breast cancer ASCs with NK cells is not clearly defined. In this study, interaction of breast ASCs with NK cells was investigated. Thus corresponding results could help better understand how breast adipose derived stem cells affect the phenotype of NK cells. 

## Materials and Methods

### Subjects

In this experimental study, ASCs were isolated from 20 Iranian breast cancer patients, 10 patients with pathological stage II and 10 with pathological stage III. Patients were not undergoing any treatment such as chemotherapy prior to obtaining breast adipose tissues. The mean and median ages of patients were 40 ± 15.4 and 41 years, respectively. The data of breast cancer ASCs were compared to ASCs isolated from 10 normal women who had undergone cosmetic mamoplasty surgery. The mean and median ages of normal individuals were 31 ± 4 and 30 years, respectively. Sample collection was carried out after obtaining approval of the Ethics Committee, Shiraz University of Medical Sciences, Shiraz, Iran. All participants fulfilled informed consent form before contribution in this study. 

## Adipose derived stem cells isolation and culture

ASCs were isolated from breast adipose tissues as described previously (20). Briefly, fragments of adipose tissue were digested with 0.2% collagenase type I (Gibco, USA). They were then centrifuged and the stromal vascular fraction (SVF) was isolated using Ficoll-Paque (Biosera, UK). The SVF pellet was suspended in DMEM culture medium (Gibco, USA) containing 10% fetal bovine serum (FBS, Gibco, USA) and 1% penicillin/streptomycin (Biosera, UK) in 25 cm^2^ tissue-culture flasks, followed by incubation at 37˚C in a 5% humidified CO_2_ incubator. Nonadherent cells were discarded after 24 hours culturing and fresh medium was added. The adherent cells were cultured by changing medium every 4 days and harvested in passage 3 or 4 nearly after 30 days culture. After 3-4 days individual cell colonies were visible.

## Separation and culture of peripheral blood lymphocytes

5-10 milliliters of peripheral blood was obtained from healthy donors and gently added to the same volume of Ficoll-Paque and centrifuged 15 minutes for density gradient separation of peripheral blood mononuclear cells (PBMCs). The ring of PMBCs was transferred into a tube and washed with phosphate buffer saline (PBS). Isolated PBMCs were cultured for 2 hours in order to exclude monocytes from mononuclear cells. After incubation time, the remaining flout cells (PBLs) were collected. Then, in order to obtain short-term polyclonal activation, PBLs were cultured in MLA-144 cell line supernatant, as a source of IL-2, diluted with RPMI culture medium (Biosera, UK) containing 10% FBS for 24 hours. 

## Culture of breast cancer and normal adipose derived stem cells with peripheral blood

lymphocytes and phenotypic analysis of natural killer cells PBLs were cultured with ASCs at 5:1 PBLs/ ASCs, as a suitable cell ratio to obtain ASCmediated inhibition of NK-cell proliferation, in a transwell coculture system (BD, USA) for 72 hours. The control was activated PBLs with MLA144 supernatant, in absence of ASCs in culture. After 72 hours, PBLs and ASCs were assessed through flow-cytometry and quantitative reverse transcriptase-polymerase chain reaction (qRTPCR), respectively. For flow-cytometric analysis, PBL cells were collected, washed twice with PBS and stained with fluorescent-labeled monoclonal antibodies (mAbs). The following mAbs were used: PE-conjugated anti-CD16, anti-NKp30, anti-NKp44, anti-NKG2A, FITC-conjugated anti-CD3, anti-CD16, anti-CD69, APC-labeled anti-CD3 and Percp-labeled anti-CD56; positive cells were counted and compared to the signals of the corresponding antibody isotype controls. All antibodies were provided from BD Bioscience (USA). Approximately 100,000 events were collected on a four colors Becton Dickinson Fluorescence-Activated Cell Sorting (FACS) Caliber instrument and further analyzed using FlowJo software version 7.6. 

## RNA extraction and cDNA synthesis

Total RNA was extracted from ASCs using TRizol Reagent (Invitrogen, Germany). The extracted RNAs were used for preparation of cDNA using the cDNA synthesis kit based on the manufacturer’s instructions (Fermentas, Canada). 

## Quantitative reverse tramscriptase polymerase chain reaction

mRNA expressions of *IDO1*, *IDO2* and *HLA-G* were assessed in the co-cultured ASCs with PBLs by qRT-PCR method using an ABI 7500 RealTime PCR System (Applied Biosystems, USA). For that, 2 μl cDNA was amplified in 20 μl PCR mixture containing 150 nM of each forward and reverse primers [designed with primer blast online software (21)], 10 μl of 2×SYBR Green PCR Master Mix (Fermentas, Canada), and 7.4 μl diethylpyrocarbonate (DEPC) treated water. PCR amplification was carried out in 50 cycles and thermal cycling for all genes was performed through denaturation step at 95˚C for 10 minutes, 95˚C for 15 seconds, annealing at 57˚C for 30 seconds and extension at 60˚C for 1 minute. All data were compared to 18s rRNA, as housekeeping gene. 

## Statistical analysis

Expression of *IDO1*, *IDO2* and *HLA-G* mRNAs in ASCs were determined using 2^-ΔΔCT^method. A P value of less than 0.05 was statistically considered significant for mRNA expression, pathological information of the patients, and data of flow-cytometry using the Mann-Whitney nonparametric U-test. Graphs were presented using GraphPad Prism 5 software. 

## Results

### Expressions of *IDO1*, *IDO2* and *HLA-G5* in adipose derived stem cells of breast cancer patients compared to normal individuals 

**Fig.1:**
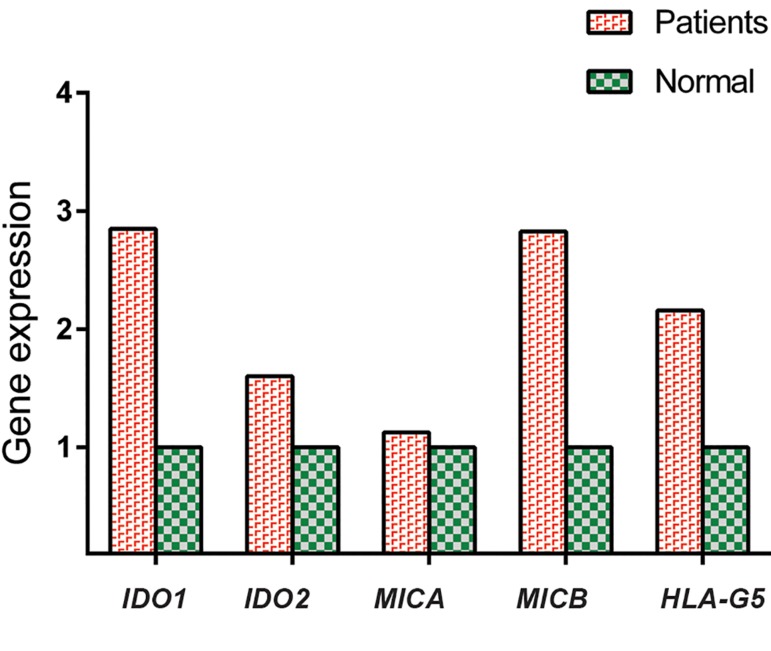
Relative quantifications of *IDO1*, *IDO2*, and *HLA-G* in adipose derived stem cells (ASCs) of breast cancer patients and controls. Data were shown as the median of gene expression. P>0.05 for all genes.

mRNA expression of *IDO1*, *IDO2*, and *HLA-G5* in ASCs of both patients (n=20) and controls (n=10) are shown in Figure 1. mRNA expression of *IDO1*, *IDO2* and *HLA-G5* in breast cancer patients were respectively 2.9, 1.6 and 2.2 fold more than normal individuals, although these differences were not statistically significant (P=0.17, 0.85 and 0.81, respectively). 

**Fig.2:**
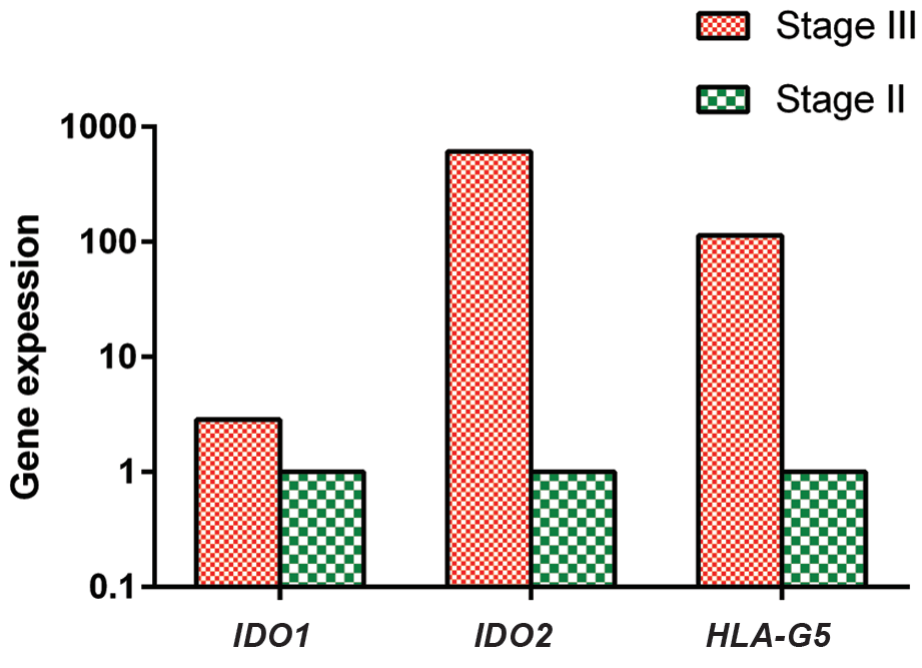
Relative quantifications of *IDO1*, *IDO2* and *HLA-G5* in adipose derived stem cells (ASCs) of breast cancer patients with pathological stage II and stage III tumors. Data were shown as the median of gene expression. P>0.05 for all genes.

In addition, mRNA expressions of *IDO1*, *IDO2* and *HLA-G5* in breast cancer patients with pathological stage III were respectively 2.9, 607 and 113.7 fold more than ASCs compared to stage II, however this differences were not statistically significant ( P=0.32, 0.14 and 0.17, respectively, (Fig.2). 

### Expressions of *IDO1*, *IDO2* and *HLA-G5* in adipose derived stem cells of breast cancer patients with pathological stage III tumors before and after co-culturing with peripheral blood lymphocytes 

**Fig.3:**
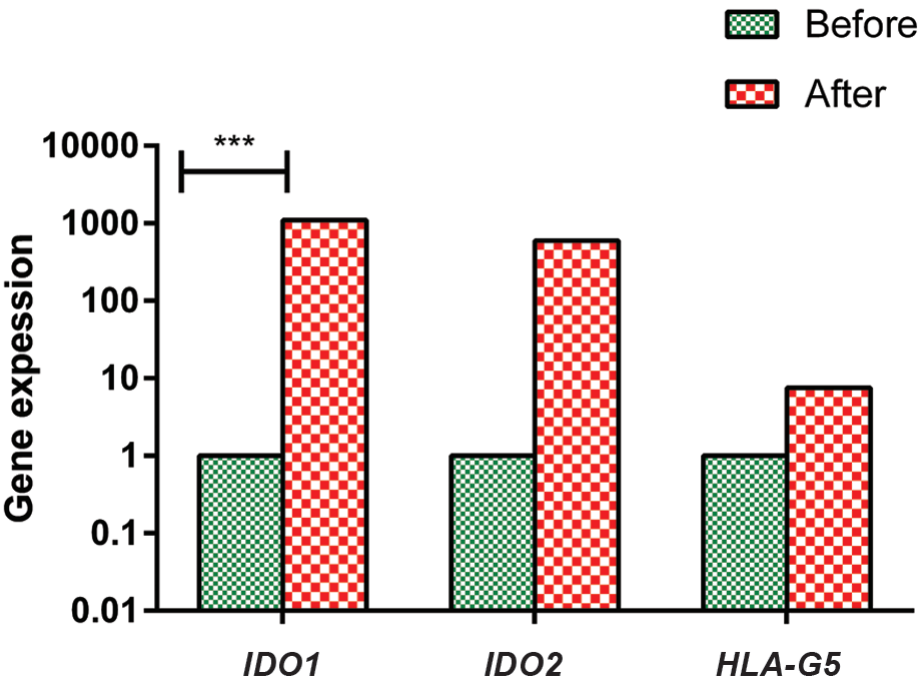
Relative quantifications of *IDO1*, *IDO2*, and *HLA-G5* in adipose derived stem cells (ASCs) of breast cancer patients with stage III before and after co-culturing with PBLs. Data were shown as the median of gene expression. ***; P<0.001.

As shown in Figure 3, mRNA expressions of *IDO1*, *IDO2* and *HLA-G5* in ASCs of patients with pathological stage III tumors after co-culturing with PBLs were respectively 1100, 600 and 7.5 fold more than ASCs, before co-culturing with PBLs. The differences were statistically significant only for *IDO1* mRNA expression (P=0.0002). 

### Changes in natural killer cell subpopulations after exposure of peripheral blood lymphocytes to breast cancer and normal adipose derived stem cells 

**Fig.4:**
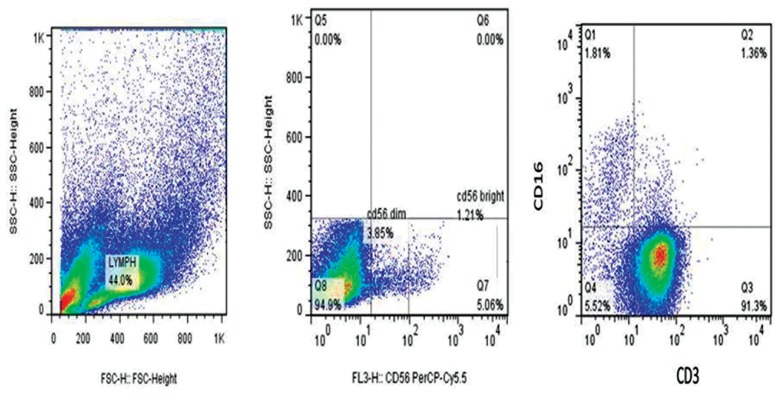
The schematic analysis of natural killer (NK) cell subpopulations among peripheral blood lymphocytes (PBLs). SSC versus FSC density plot, SSC versus PerCP-Cy5.5 and APC-labeled anti-CD3 versus FITC conjugated anti-CD16 fluorescence density plot.

To analyze possible interactions between NK cells and ASCs, we initially assessed the properties of NK cell subpopulations, in the absence or presence of ASCs from 5 normal individuals and 5 patients with pathological stage III tumor, by flow-cytometric analysis using anti-CD3, CD16 and CD56 specific antibodies. Before experiment, PBLs were activated with MLA-144 conditioned media for 48 hours. For flow-cytometric analysis, lymphocytes were gated for CD3^-^CD16^+^cells, then the cell population was evaluated for CD56 expression. As shown in Figure 4, based on the expression of CD56, NK cells were divided into two subpopulations: CD56^dim^ and CD56^bright^cells. To compare the effect of ASCs on different subpopulations of NK cells, these subpopulations were assessed in the absence of ASCs and in the presence of either cancer ASCs with stage III or normal ASCs. Accordingly, no statistically significant difference was found in the percentages of CD3^-^CD16^+^ CD56^dim^and CD3^-^CD16^+^CD56^bright^cells between different conditions (P=0.4 and 0.17, respectively).

**Fig.5:**
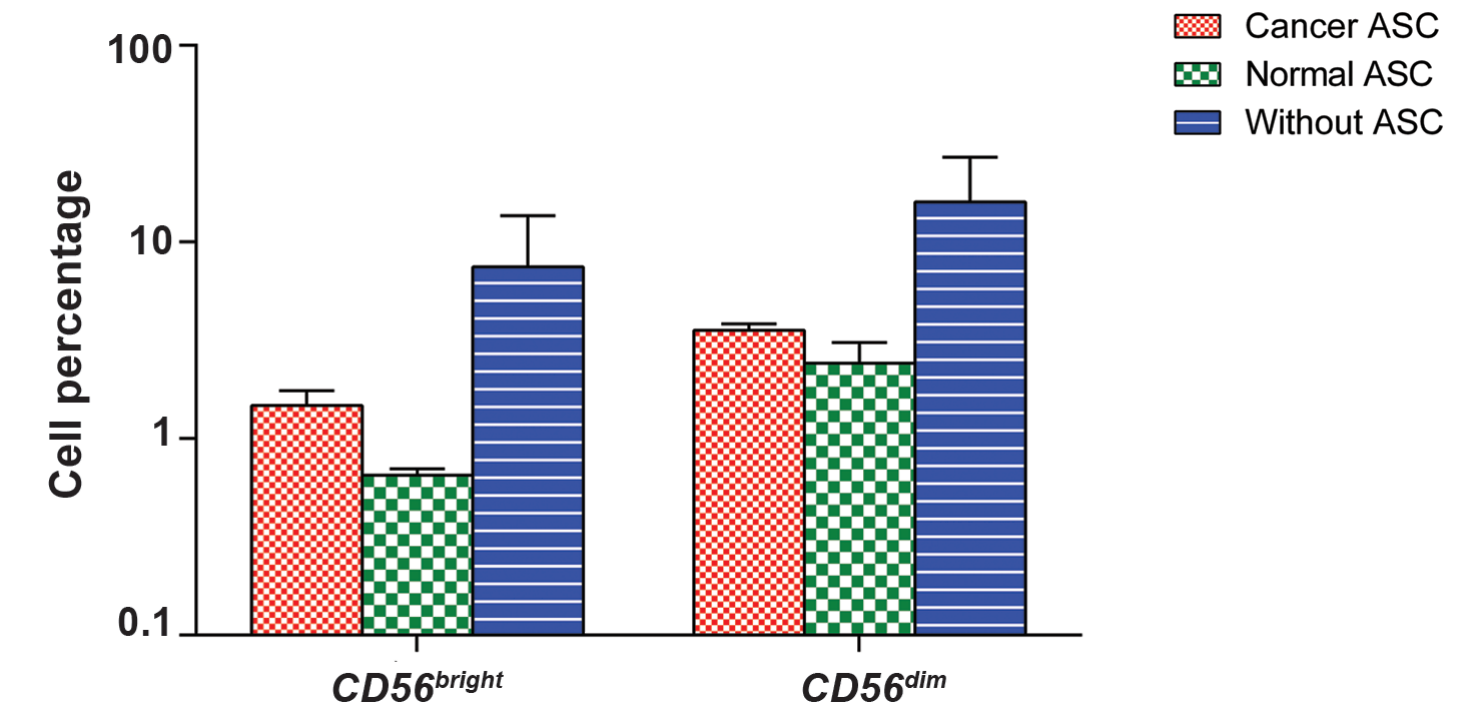
Comparison of different populations of natural killer (NK) cells in the presence of cancer adipose derived stem cells (ASCs), normal ASCs and without ASCs.

As depicted in Figure 5, the percentage of both cell subsets was decreased after exposure of PBLs to either breast cancer or normal ASCs. The CD3^-^CD16^+^ CD56^bright^cell percentage was 7.48 ± 6.11, 0.65 ± 0.05 and 1.47 ± 0.28 in respectively unexposed PBLs, PBLs+normal ASCs and PBLs+cancer ASCs. In fact, CD3^-^CD16^+^ CD56^bright^cells showed 6.6 and 4.5 fold lower percentage when exposed to normal and cancer ASCs, respectively, compared to unexposed PBLs. The CD3^-^CD16^+^ CD56^dim^cell percentage was 16.01 ± 10.9, 2.41 ± 0.67 and 3.56 ± 0.27 in unexposed PBLs, PBLs+normal ASCs and PBLs+cancer ASCs, respectively. Hence, CD3^-^CD16^+^CD56^dim^cells showed 11.5 and 7 fold lower percentages when exposed to normal and cancer ASCs, respectively, compared to PBLs cultured alone. These differences were not statistically significant (P>0.05).

### Adipose derived stem cells affect the expression of activating and inhibitory receptors of natural killer cells 

**Fig.6:**
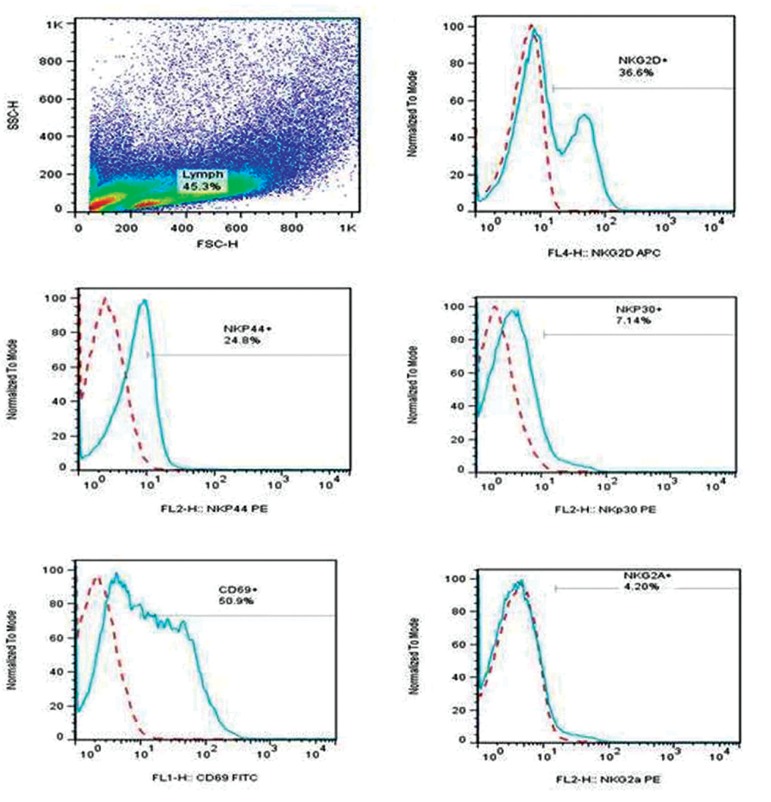
SSC versus FSC density plot and the histogram representations of NKG2D, NKp44, NKp30, CD69 and NKG2A on natural killer (NK) cells by flow-cytometric analysis. Solid profiles represent expression of receptors and dashed histograms represent negative control.

**Fig.7:**
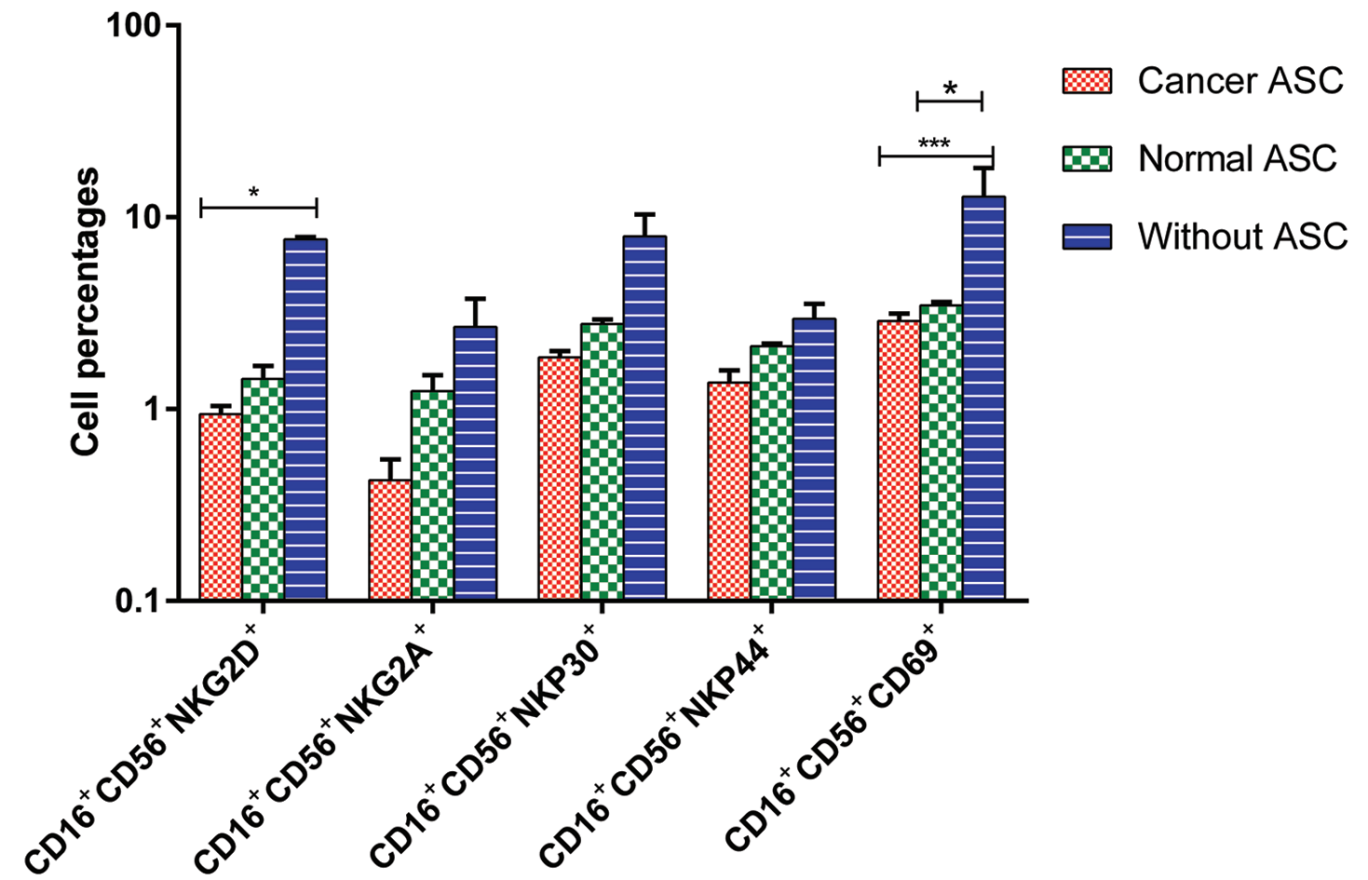
Expression of activating and inhibitory natural killer (NK) receptors in the presence or absence of cancer or normal adipose derived stem cells (ASCs). Data were shown as mean ± SEM of cell percentages. *; P<0.05 and ***; P<0.01.

In order to determine the effect of ASCs on PBL derived NK cells, we assessed NK cells for the expression of activating and inhibitory receptors in different conditions (in the presence of breast cancer and normal ASCs, with respect to PBLs cultured without ASCs). We performed flow-cytometric analysis using mAbs specific for activating and inhibitory NK receptors including CD69, NKp30, NKp44, NKG2D and NKG2A. The schematic representation of flow-cytometer analysis for the expressions of different NK receptors is shown in Figure 6. As represented in Figure 7, no significant change was observed in NKp30^+^, NKp44^+^and NKG2A^+^cells in the presence or absence of ASCs. However, the differences were statistically significant for NKG2D^+^cells between PBLs cultured alone and the same cells cultured with breast cancer ASCs (P<0.05) and for CD69^+^cells between unexposed PBLs versus those cultured with breast cancer and normal ASCs (P<0.01 and <0.05, respectively). The mean ± SEM of NKG2D^+^cells were 1 ± 0.1, 1.4 ± 0.2 and 7.7 ± 0.2 in the presence of cancer and normal ASCs compared to PBLs alone, respectively. The mean ± SEM of CD69^+^cells were 2.9 ± 0.3 in the presence of cancer ASCs, 3.5 ± 0.1 in the presence of normal ASCs and 12.8 ± 5 in PBLs cultured alone.

## Discussion

In the present study, we compared breast cancer and normal ASCs for the expression of inhibitory molecules and the effect of these cells on the phenotype of NK cells. Results provide evidence that breast cancer derived ASCs are different from normal ASCs in the expression of inhibitory molecules such as *IDO* and *HLA-G5* and this type of stem cell can exert inhibitory effects on the expression of activating NK receptors. 

Recently, it has been demonstrated that MSCs have inhibitory effects on almost all types of immune cells. They are capable to inhibit differentiation and maturation of dendritic cells (15), to arrest T-cell activation (22, 23), and to reduce production of inflammatory cytokines by different immune cells (16). It has been reported that MSCs inhibit proliferation of resting NK cells, and also NK mediated cytotoxic activity and IFNγ production (24, 25). The molecular basis of such inhibitory effect is not well defined but Spaggiari et al. reported an inhibition of the surface expression of NKp30 and NKG2D, while no surface expression of the NKp44 activating receptor was observed in NK cells cultured with bone marrow derived MSCs (BM-MSCs). They also showed a strong inhibitory effect of MSCs on proliferation, cytotoxic activity and production of cytokines by NK cells (26).

Consistently, presence of ASCs (as a kind of mesenchymal stem cell) in our experimental culture condition decreased the percentage of both CD3^-^CD16^+^ CD56^bright^ and CD3^-^CD16^+^ CD56^dim^ cell subsets after exposure of PBLs to either breast cancer or normal ASCs. Although the presence of ASCs had no significant effect on the percentage of NKp30^+^, NKp44^+^and NKG2A^+^NK cells, it caused considerable reduction in the NKG2D^+^and CD69^+^NK cells among the cultured PBLs with breast cancer or normal ASCs versus the same cells cultured alone. No significant difference was observed between the effect of breast cancer and normal ASCs.

The inhibitory effect of ASCs on NK cells may reflect production of soluble factors. As shown in our previous experiments, breast derived ASCs, especially breast cancer ASCs, express IL-10 and TGF-β1. In addition, the culture supernatant of ASCs isolated from breast cancer patients with pathological stage III had an ability to induce different cytokines such as IL-4, TGF-β1 and IL-10 in PBLs and to upregulate CD4^+^CD25^high^ Foxp3^+^ T regulatory cells (20). Mesenchymal stem cells gain the ability to produce a wide range of modulators such as IL-15, TGF-β1, PGE2 and IDO which results in important effects on different types of immune cells such as NK cells (27, 28). Expression of IDO in the tumor microenvironment negatively affects the immune response within the tumor through stimulating a regulatory phenotype in CD4^+^ T cells (29, 30) as well as reduction in the infiltration of CD3^+^and CD8^+^ T-lymphocyte and CD57^+^ NK cells (31). The synergistic effect of PGE2 and IDO in BM-MSC mediated inhibition of NK cells has also been reported (26). High levels of IDO have been detected in advanced stages of ovarian carcinoma and nasopharyngeal carcinoma (32, 33). IDO has the ability to show immunosuppressive effects by increasing the infiltration of Foxp3+ regulatory T cells in patients with breast cancer (34). It was reported that expression of IDO is associated with clinical stage and lymph node metastasis of breast cancer (35).

Here we showed that both breast cancer and normal ASCs can produce regulatory molecules such as *IDO1*, *IDO2*, and *HLA-G5*. Interestingly all molecules had higher expression of mRNA in breast cancer versus normal ASCs and ASCs from patients with pathological stage III compared to stage II. In order to see whether PBLs can modify the expression of regulatory molecules, expression of *IDO1*, *IDO2* and *HLA-G5*s were assessed in ASCs before and after coculture with PBLs. Based on the results, expression of all molecules, particularly *IDO1* in ASCs, were significantly increased after exposure of ASCs to PBLs. 

It is plausible because it has been demonstrated that some mediators such as PGE2 can induce the expression of *IDO* (36). It has been shown that MSCs gain the ability to synthesize IDO under the influence of NK cell derived IFNγ and TNFα or through autocrine stimulation of cells by PGE2 (26). Therefore, it seems that a vicious cycle exists between residential mesenchymal stem cells and recruiting immune cells surrounding tumor cells. If it is true, MSCs must be considered as important players in the tumor microenvironment since their inhibitory effects may modify the destiny of tumor cells. 

The most important advantage of our study is the comparison of ASC effects from cancer patients with normal controls while other reports only showed the cross talk between MSCs from normal individuals and NK cells. On the other hand, low number of patients in each stage as well as isolating no NK cells from PBLs were the most important limitations of this study. 

## Conclusion

The present data provides evidences for the ASC-NK cell interaction and the inhibitory effects of ASCs on NK cells through significant reduction of activating NK receptors such as NKG2D and CD69. Therefore, more evidences for the immunosuppression of ASCs on NK cells are obtained, providing conditions in favor of tumor immune evasion. Accordingly, these cells are better to be considered in therapeutic interventions of cancer therapy. 
